# Whole Cell Formaldehyde Cross-Linking Simplifies Purification of Mitochondrial Nucleoids and Associated Proteins Involved in Mitochondrial Gene Expression

**DOI:** 10.1371/journal.pone.0116726

**Published:** 2015-02-19

**Authors:** Nina Rajala, Fenna Hensen, Hans J. C. T. Wessels, Daniel Ives, Jolein Gloerich, Johannes N. Spelbrink

**Affiliations:** 1 Mitochondrial DNA Maintenance Group, BioMediTech, FI-33014 University of Tampere, Tampere, Finland; 2 Department of Pediatrics, Nijmegen Centre for Mitochondrial Disorders, Radboud University Medical Centre, Geert Grooteplein 10, P.O. Box 9101, 6500 HB, Nijmegen, The Netherlands; 3 Radboud Proteomics Centre, Department of Laboratory Medicine, Laboratory of Genetic Endocrine and Metabolic Disorders, Radboud University Medical Centre, Geert Grooteplein 10, P.O. Box 9101, 6500 HB, Nijmegen, The Netherlands; 4 MRC-National Institute for Medical Research, Mill Hill, London, United Kingdom; Osaka University, JAPAN

## Abstract

Mitochondrial DNA/protein complexes (nucleoids) appear as discrete entities inside the mitochondrial network when observed by live-cell imaging and immunofluorescence. This somewhat trivial observation in recent years has spurred research towards isolation of these complexes and the identification of nucleoid-associated proteins. Here we show that whole cell formaldehyde crosslinking combined with affinity purification and tandem mass-spectrometry provides a simple and reproducible method to identify potential nucleoid associated proteins. The method avoids spurious mitochondrial isolation and subsequent multifarious nucleoid enrichment protocols and can be implemented to allow for label-free quantification (LFQ) by mass-spectrometry. Using expression of a Flag-tagged Twinkle helicase and appropriate controls we show that this method identifies many previously identified nucleoid associated proteins. Using LFQ to compare HEK293 cells with and without mtDNA, but both expressing Twinkle-FLAG, identifies many proteins that are reduced or absent in the absence of mtDNA. This set not only includes established mtDNA maintenance proteins but also many proteins involved in mitochondrial RNA metabolism and translation and therefore represents what can be considered an mtDNA gene expression proteome. Our data provides a very valuable resource for both basic mitochondrial researchers as well as clinical geneticists working to identify novel disease genes on the basis of exome sequence data.

## Introduction

Mammalian mitochondrial DNA (mtDNA) was discovered in the 1960´s[1,2] and early studies in 1969 by Nass suggested that mtDNA could be membrane bound[3]. Later studies postulated that mtDNA is attached to the inner membrane involving the major non-coding D-loop region[4,5]. The first microscopic observation of mtDNA as discrete structures within mitochondria came from the use of a DNA stain in the yeast *S*. *cerevisiae*. However, despite this evidence and many additional studies in yeast and many other, often vertebrate, species (see below), mtDNA in mammals was for many years described as naked. This view has changed over the last 15–20 years and mtDNA is now generally considered to be organized in discrete nucleo-protein complexes that are designated nucleoids by analogy to nucleo-protein complexes in bacteria[6,7]. Studies in *Xenopus laevis* oocytes suggested that mtDNA was packaged in a compact beaded structure that was membrane associated[8]. Mignotte & Barat[9] characterised a single 28 kDa protein component of the “beads” that was able to introduce superhelical turns, later identified as mitochondrial transcription factor A (TFAM)[10]. TFAM (Abf2 in yeast) is considered to be the principle mtDNA packaging factor[7]. Mitochondrial single stranded DNA binding protein (mtSSB) was also one of the early identified nucleoid proteins[11]. Twinkle, the mitochondrial DNA helicase, was the first mammalian protein shown to co-localise with mtDNA in immunofluorescence studies[12] and is part of a minimal replisome together with mtSSB and Polymerase gamma (POLG) in *in vitro* studies[13]. TFAM and mtSSB were shown also to co-localise with mtDNA *in situ*[14–16], the latter showing enrichment in particular with replicating nucleoids[17].

The above proteins (and where conserved, their yeast counterparts) are all considered *bona fide* nucleoid associated proteins (NAPs) and have a clear function in mtDNA packaging, replication and transcription. However, it has become clear that many additional factors associate with mtDNA to facilitate mtDNA maintenance as well as gene expression[18–26]. In particular in yeast, these factors have been show to associate both transiently and under specific metabolic conditions[27]. By comparison of yeast and vertebrate nucleoid proteomes it has also become clear that there appears to be little conservation of associated additional factors. This is considered a consequence of divergent protein-mtDNA co-evolution[28].

In order to fully understand mammalian mtDNA maintenance and gene expression, and solve conflicting models for example for mtDNA replication, the identification and functional study of the full set of proteins involved in mtDNA metabolism is important. One approach to identify NAPs is via biochemical isolation and mass spectrometric identification. Over the last 10 years various sets of NAPs were identified, but as we discussed recently, few proteins are shared between all these sets[29], a consequence of the various methods and starting materials employed, the stringency of isolation, the target at which isolation was directed and the fact that many protein-nucleoid interactions are transient in nature. On the basis of this comparison we also concluded that the most inclusive method, identifying most factors known to interact with mtDNA involved a formaldehyde cross-linking step. However, very few of the studies published so far have used quantitative proteomics and typically have presented the data of just one or two purifications (see [29]). This low replicate number is probably due to the complexity of some of the isolation procedures involved that require large quantities of starting material.

Here we present the shotgun proteomics results using a greatly simplified mtDNA nucleoid proteomics analysis using whole cell formaldehyde cross-linking followed by cell lysis and affinity purification. Here, induced overexpression of a FLAG-tagged mtDNA helicase Twinkle was used because overexpressed Twinkle specifically co-localizes with mtDNA nucleoids[17] and short, low level induction was previously shown to minimally impact on nucleoid structure, mtDNA levels and transcription[30,31]. Because we carry out the cross-linking in whole cells, this eliminates the need to isolate mitochondria and use subsequent nucleoid purification steps. By comparing non-cross-linked with cross-linked Twinkle-FLAG samples versus non-cross-linked and cross-linked control cells that express a mitochondrially targeted and FLAG-tagged Luciferase we show that many previously identified nucleoid proteins were specifically enriched in cross-linked Twinkle-FLAG purification. Here, the comprehensive use of both non cross-linked and cross-linked samples and controls in combination with multiple biological and technical repeats by accurate label free quantification (LFQ) provides a firmer basis for the consideration of many putative NAPs and identifies an inclusive list of proteins not just for mtDNA maintenance but also for mitochondrial RNA metabolism and translation. In addition, we identified several potential new NAPs. Finally, in a comparison of Twinkle-FLAG expressing cells either or not containing mtDNA we identify those proteins that co-purify with Twinkle-FLAG because of the presence of mtDNA/RNA, which suggests that many of these proteins interact with mtDNA/RNA but not directly with Twinkle. The ease of our method and application of LFQ is expected to find much wider application in the study of dynamic mitochondrial protein-protein and protein-nucleic acid interactions.

## Materials & Methods

### Routine cell culture and creation and maintenance of stable transfected inducible expression cell lines

Stable cell lines expressing various mtDNA maintenance proteins upon induction were created as described[30] using the Flp-In T-Rex 293 host cell line (*Invitrogen*), a HEK293 variant containing a Flip recombination site at a transcriptionally active locus, or Flp-In T-Rex 293 ρ° cells (see below). The resulting cells were grown in DMEM medium (*Sigma*) supplemented with 10% FCS (*Sigma*), 2 mM L-glutamine, 1 mM Na-pyruvate, and with the addition of 50 μg/ml uridine (*Sigma*) in ρ° cells, 100 μg/ml Hygromycin and 15 μg/ml Blasticidin (*Invivogen*) in a 37°C incubator at 8.5% CO2. Flp-In T-Rex 293 expressing a mitochondrially targeted and FLAG tagged Luciferase (mtLucFLAG) were a kind gift of Profs. Robert Lightowlers and Zosia Chrzanowska-Lightowlers (see also[32]).

To isolate a ρ° variant of the HEK293 Flp-In T-Rex cell line, cells were grown for an extended period of time in standard medium supplemented with 50 ng/ml Ethidium Bromide (EB) and 50 μg/ml uridine. EB treated HEK293 Flp-In T-Rex cells were tested for mtDNA depletion by growth on galactose medium. Galactose medium contained glucose-free DMEM, 1 mM (0.5 mg/l) pyruvate and 5 mM (0.9 mg/ml) filter-sterilised D-(+)-galactose (*Sigma*). Cells were further tested for total mtDNA depletion by southern blot of total DNA with D-loop (H1) probe (S1 Fig.). This result suggested that prolonged EB treatment had successfully depleted HEK293 Flp-In T-Rex cells of their mtDNA. This was confirmed when the putative ρ^0^ cells were grown in the absence of EB for a period of several months and still found to lack any detectable mtDNA by dot-blot analysis (unpublished data Ş. Cansız-Arda and J.M. Gerhold, Spelbrink lab). Prior to southern blot, total DNA was extracted by isoproponal precipitation, digested overnight with *Pvu*II at 37°C, heat denatured at 70°C for 10 minutes and separated on a 1.0% TBE agarose gel at room temperature for 3 hours at 100 volts. D-loop (H1) probe (16241–141) primers: Forward – TTACAGTCAAATCCCTTCTCGT, Reverse – GGATGAGGCAGGAATCAAAGACG.

### Western blot analysis

Immunoprecipitation eluates were analysed for proteins by immunoblotting after SDS–PAGE[33]. Antibody dilutions were as follows: primary FLAG monoclonal (*Sigma*), 1:4000, TFAM rabbit polyclonal antibody (kind gift of Dr. R. Wiesner), 1:10000; Twinkle mouse monoclonal (kind gift of Anu Wartiovaara-Suomalainen) 1:1000; mtSSB rabbit polyclonal (Sigma, HPA002866), 1:2000; POLG1 goat polyclonal (*Santa Cruz*, sc-5931), 1:1000. Peroxidase-coupled secondary antibody horse-anti-mouse or goat-anti-rabbit (*Vector Laboratories*) 1:5000.

### Formaldehyde cross-linking and immunoprecipitation

Twinkle expression was induced by addition of 3ng/ml doxycycline (*Sigma*) for 36 hours. From previous experiments we know that this expression level and time is appropriate to preserve nucleoid structures[31]. For cross-linking typically cells from five 145 mm (cross-section) cell culture dishes were harvested and cell number was adjusted to 10×10^6^ cells/ml. Cross-linking was carried out in 1% formaldehyde (*Sigma*) for 10 min at RT with rotation. The reaction was stopped by addition of 125 mM glycine, pH 8.0. Formaldehyde is toxic and was handled in a fume hood. Sample handling after addition of formaldehyde similarly was carried out in a fume hood and formaldehyde disposed appropriately. Cells were transferred on ice and all subsequent centrifugations carried out at +4°C. Cells were washed four times with ice cold TBS (50 mM Tris-HCl pH 7.4, 150 mM NaCl) and processed further by two different methods. Method A, Triton X-100 method: Cells were lysed in Buffer A (50mM Tris-HCl pH 7.4, 300 mM NaCl, 2mM EDTA 1% Triton X-100). In method B, the X-ChiP method, cells were lysed with RIPA buffer (50mM Tris-HCl pH 8, 150mM NaCl, 1% NP-40 (Igepal), 0.5% sodium deoxycholate, 0.1% SDS). In both methods lysates were sonicated for 1 min at 40% power (1s on 2s off cooling on ice), but only with the X-ChiP method sonication was followed by addition of 100μg/ml RNAse A (*Sigma*), 5U/ml DNAse I (*Thermo Scientific*) and 50U/ml Benzonase nuclease (*Sigma*), 2.5mM Mg2+, 1mM CaCl2 and incubated at +37°C for 30min. With both methods lysates were centrifuged for 10 min at 1200g at +4°C and the protein content of the lysates was equalised to 2mg/ml in a total volume of 10 ml before addition of 180 μl of FLAG resin (*Sigma*) and rotation for 2 hours at +4°C. In method A, FLAG resin was washed once in buffer B, C and D. Buffer B: 50mM Tris-HCl pH 7.4, 800mM NaCl, 0.1% Triton-X 100, Buffer C: 50mM Tris-HCl pH 7.4, 50 mM NaCl, Buffer D: 50mM Tris-HCl pH 7.4, 150 mM NaCl, 0.1% Triton-X100. Nucleoids were eluted with 100 μl 3xFLAG peptide (at 0.25 mg/ml) in 50mM Tris-HCl pH 7.4, 150 mM NaCl. In method B, the FLAG resin was washed three times in RIPA buffer and nucleoids eluted with 100 μl 3xFLAG peptide (at 0.25 mg/ml) in RIPA buffer. All buffers included 1×complete EDTA-free Protease inhibitors (*Roche*).

### Mass spectrometry sample preparation

Protein samples were incubated with SDS-PAGE sample-buffer for 30 min at 95°C to reverse FA cross-links and fractionated by SDS-PAGE on Any kD Mini-PROTEAN TGX Gels (*BIO-RAD*). Lanes were cut in in three equal-sized (approximately 1x2.5 cm) gel slices. No gel-staining was applied following electrophoresis. Each gel slice was subjected to *in-gel* tryptic digestion and further processed according to standard methods[34]. In short, gel slices were cut into small pieces (~1mm^2^) and were washed successively at least three times with 50 mM ammonium bicarbonate (ABC) and 100% acetonitrile (ACN). Gel slices were swelled in 10 mM dithiothreitol and incubated for 20 minutes at 56°C to reduce protein disulfide bonds. To remove the reduction buffer, gelpieces were shrunk with ACN. Alkylation of the reduced cysteines was performed by incubation of 50mM chloroacetamide in ABC for 20 minutes at room temperature in the dark. Gel pieces were again washed twice with ACN and ABC before tryptic digestion at 37°C overnight with 1.25ng/μl sequencing grade modified Trypsin (*Promega*) in ABC. To recover tryptic peptides from the gel pieces, they were first diluted 1:1 with 2% trifluoric acid (TFA), sonicated for 30 seconds, and incubated at RT for ≥ 15 minutes with gentle agitation. Supernatant was transferred to a fresh tube and the gel pieces were shrunk with 100% ACN at RT at gentle agitation for ≥ 15 minutes to recover remaining peptides from the gel. Supernatant was pooled and subjected to vacuum centrifugation to remove the ACN and concentrate the sample. Thereafter, the peptide sample was desalted and concentrated by “STop And Go Extraction (STAGE) tips”[35].

### Mass spectrometric measurements

Measurements were performed by nanoflow reversed-phase C18 liquid chromatography (EASY nLC, *Thermo Scientific*) coupled online to a 7 Tesla linear ion trap Fourier-Transform ion cyclotron resonance mass spectrometer (LTQ FT Ultra, *Thermo Scientific*) or by nanoLC 1000 (*Thermo Scientific)* chromatography coupled online to Q Exactive hybrid quadrupole-Orbitrap mass spectrometer (*Thermo Scientific)*. Chromatography was performed with an Acclaim PepMap 0.3 x 5 mm 5μm 100Å trap column (*Thermo scientific*) in combination with a 15cm long x 100μm ID fused silica electrospray emitter (*New Objective*, PicoTip Emitter, FS360-100-8-N-5-C15) packed *in-house* with ReproSil-Pur C18-AQ 3 μm 140Å resin (*Dr*. *Maisch*)[36]. Tryptic peptides were loaded onto the trap column using 0.1% formic acid and separated by a linear 60 minutes (LTQ-FT) or 30 minutes (Q Exactive) gradient of 5–35% acetonitril containing 0.1% formic acid at a flow rate of 300 nl/min. For the LTQ-FT; the mass spectrometer was set to positive ion mode and acquired one full MS survey scan in the ICR cell parallel to up to four data dependent collision induced dissociation (CID) fragmentation spectra by the linear ion trap. Full MS precursor scans were performed with a single microscan at 100.000 resolving power (FWHM) at *m/z* 400 using 1E6 ions or after 2500ms injection time if this came first. Data dependent acquisition of MS/MS spectra by the linear ion trap was performed on 3E4 ions or after 750 ms maximal injection time. Fragmentation of the precursor ion by CID was performed at 30% normalized collision energy for 30 ms and activation Q = 0.25. An isolation width of 3 Th was set to isolate the precursor ion for MS/MS sequencing events. For the Q Exactive; the mass spectrometer was again set to positive ion mode. Full MS events were performed at 70.000 resolving power (FWHM) at *m/z* 200 using 1E6 ions or after 20ms of maximal injection time. Data-dependent MS/MS spectra were performed using 1E5 ions at 17.500 resolving power (FWHM) at m/z 200 or after 50ms maximal injection time for the top 10 precursor ions with an isolation width of 4.0 Th and fragmented by higher energy collisional dissociation (HCD) with a normalized collision energy of 30%.

### Mass spectrometric data analysis

Data analysis was performed with the MaxQuant software (version 1.3.0.5)[37] applying default settings with minor modifications. The precursor mass tolerance for Q Exactive measurements was set to 4.5 ppm. For both LTQ-FT and Q Exactive the multiplicity was set to 1 and Trypsin was chosen as the proteolytic enzyme allowing for 2 miscleavages. Default MaxQuant normalizations were applied. Database searches were performed on the human RefSeq database in which the reversed database is used to calculate the false discovery rate (FDR) which was set to 1% and isoleucine and leucine were forced to be treated equally. Between samples the option “Match between runs” was enabled to detect sequenced peptides which were not subjected to sequencing event in other samples and Label Free Quantification (LFQ) calculation was applied. Peptide modifications after formaldehyde cross-linking did not occur as tested by the presence of two possible modification occurring when the cross-linking is not reversed completely. The first modification is the addition of 30 Da considered to be the addition of the whole formaldehyde molecule (O = CH2) as an intermediate step in the cross-linking reaction. The second possible modification is the addition of 12 Da which equals the addition of formaldehyde followed by the release of a water molecule and is considered to be the final product [38]. Since neither modification occurred, the reversal of cross-linking seems to be complete. Furthermore, there is an increased possibility of miscleavages since the reactivity of formaldehyde is the highest on those amino acids subjected to tryptic digestion, this did not seem to give any problems since we allowed for maximum of two miscleavages and were not able to detect any miscleavage in combination with peptide modifications. Raw data files provided by MaxQuant were further analyzed manually. For the biological replicates LFQ values were used to calculate the ratios between samples per biological sample. For the triplicate measurements performed on the Q Exactive first the average LFQ values were calculated from the replicates (only proteins identified in all three replica measurements were considered), followed by calculation of the ratios between sample conditions. Whenever the ratio exceeded the value of 2 or was below 0.5, the protein was called to be respectively increased or decreased. Additional protein information such as the Gene Ontology_SLIM_cellular compartment (CC), molecular function (MF), biological process (BP) and the official gene symbol were acquired using ProteinCenter (version 3.12.10015; *Thermo Scientific*).

## Results

### Mitochondrial nucleoid proteins can be isolated following whole cell cross-linking

In order to test the applicability of whole cell XL in the analysis of mtDNA-protein nucleoid complexes we first set out to establish that we can enrich for some of the proteins associated with nucleoids using Western blot analysis. We used the inducible HEK293 FlpIn TREx system to inducibly express the mtDNA helicase Twinkle (as previously described[30,31]) with a FLAG tag at its C-terminus. Twinkle was selected as target protein since all available evidence suggests it to function as a core component of the mtDNA replisome (e.g.[13,17]).

**Fig 1:**
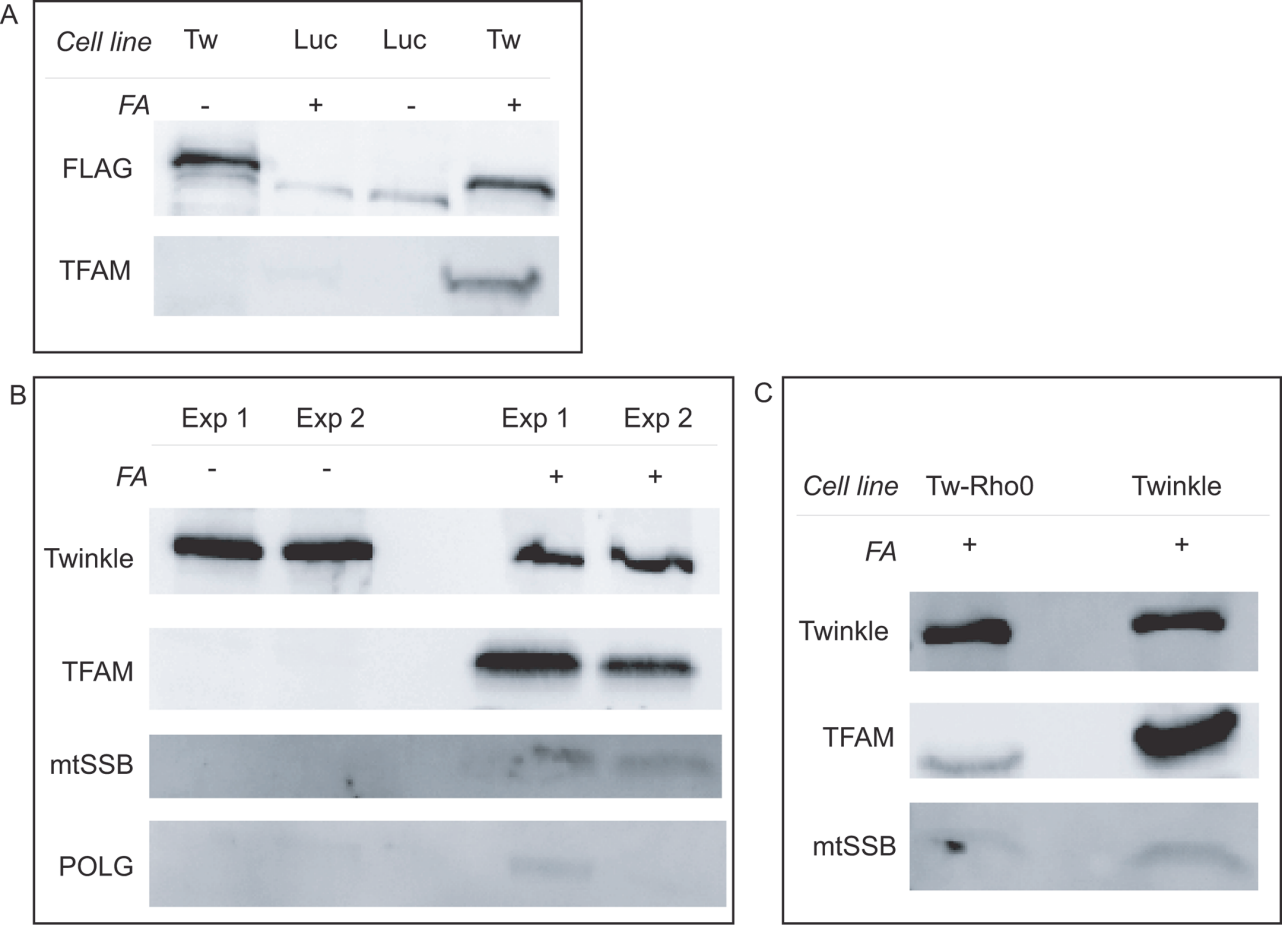
Validation of TwinkleFLAG IAP following whole cell cross-linking. HEK293 Flp-In T-Rex cells expressing either TwinkleFLAG or a mitochondrially targeted Luciferase FLAG (mtLucFLAG) were induced for 36 hrs with 3 ng/ml doxycycline, harvested, samples equalized by protein content and incubated for 10 min with 1% formaldehyde (FA) for whole cell crosslinking. Following cross-linking, cells were lysed and FLAG-tagged protein purified using FLAG immunoaffinity resin. Precipitated complexes were analysed using Western blot analysis (see *M&M* and main text for full details). Results (**A, B**) show that proteins of the mtDNA maintenance machinery are enriched with cross-linking in TwinkleFLAG expressing cells. (**C**) ρ° HEK293 Flp-In T-Rex cells expressing TwinkleFLAG were established and crosslinked samples of TwinkleFLAG expressing cells were compared with their mtDNA-containing parental cells also expressing TwinkleFLAG. Results show a very substantial decline in levels of co-purifying TFAM and mtSSB, in the absence of mtDNA.

Here, the use of tagged Twinkle overexpression over immunopurification of endogenous Twinkle was preferred because of the very low abundant endogenous expression of the protein. In addition, short Twinkle induction with a low concentration of doxycycline does not interfere with mtDNA maintenance or gene expression[30,31]. As controls we not only used parallel cultures in which no FA was applied (-XL), but also parallel cultures expressing FLAG-tagged and mitochondrially targeted Luciferase (mtLucFLAG)[32] without and with FA. Western blot analysis of these samples showed that several proteins implicated in mtDNA maintenance such as TFAM, POLG1 and mtSSB are specifically enriched by FA crosslinking in TwinkleFLAG samples, following FLAG immuno affinity purification (IAP) (Fig. 1). The results also showed that following XL, TwinkleFLAG can be affinity-purified almost as efficiently as without XL and that in principle whole cell XL in combination with IAP can be used to enrich for nucleoid associated proteins (this is validated by our mass-spectrometry analysis below). This result also shows that the FLAG tag is suitable for FA applications despite the presence of several lysine residues. Please note that these Western-blot analyses do not assess sample complexity or the percentage of mitochondrial proteins in the preparation which require mass spectrometry based methods.

### Identifying potential nucleoid associated proteins using mass spectrometry

**Fig 2:**
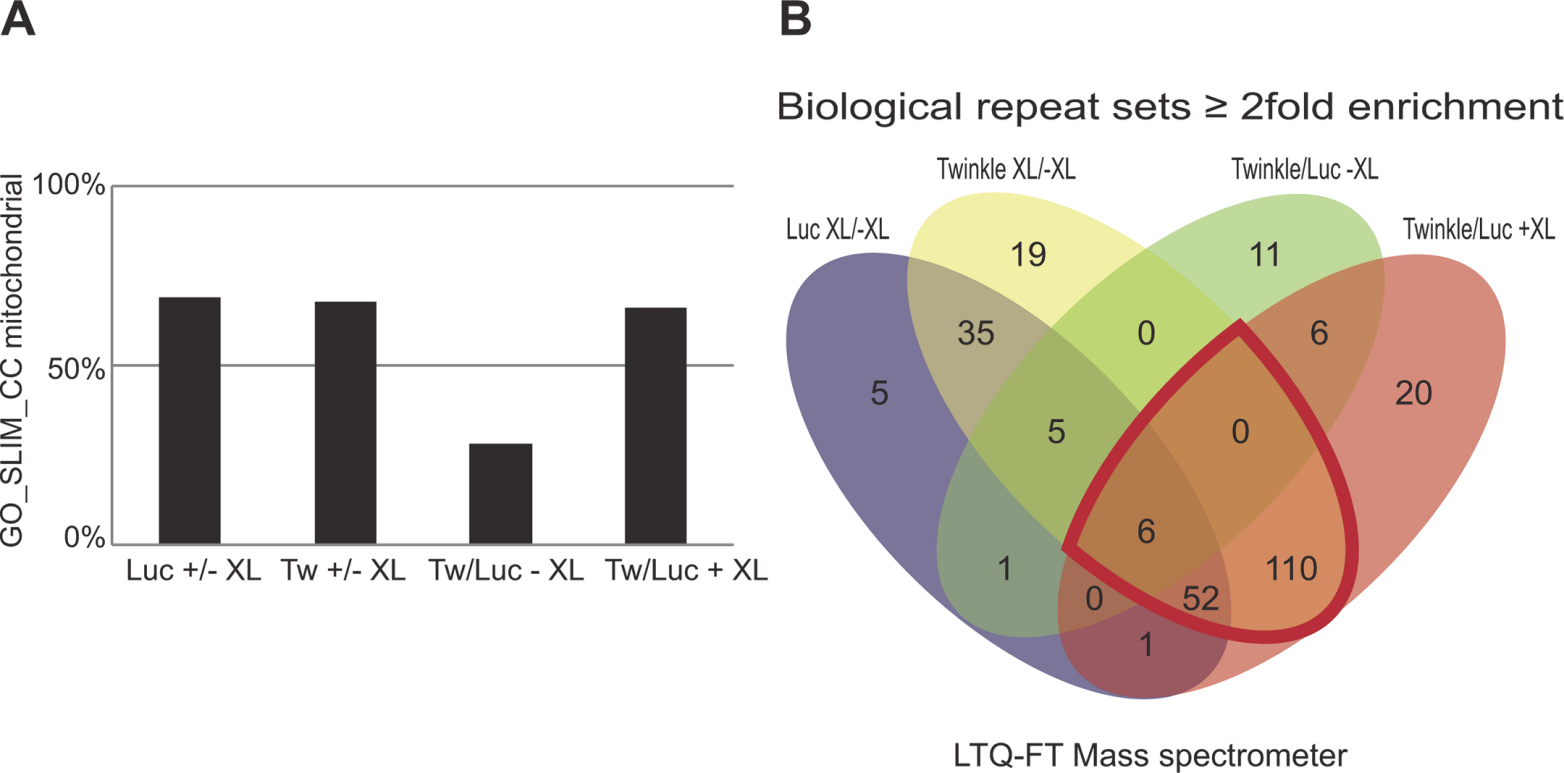
Whole cell cross-linking followed by IAP enriches for mitochondrial and nucleoid associated proteins. Protein complexes purified using FLAG-tag targeted isolation from 3 independent biological repeats using various batches of TwinkleFLAG (Twinkle) and mtLucFLAG (Luc) cells, treated either with or without FA and further processed as described in Fig. 1, were analysed by shotgun mass spectrometry. Using MaxQuant, LFQ values were derived and ratio’s calculated comparing TwinkleFLAG versus mtLucFLAG witout cross-linking (-XL) with crosslinking (+XL) as well as TwinkleFLAG +XL versus -XL and mtLucFLAG +XL versus -XL. Protein lists were compiled based on a ≥2 fold increase in LFQ values in at least 2 out of 3 experiments (see S1 Table). (**A**) Gene Ontology (GO)_SLIM_Cellular Compartment (CC) (see also *M&M*) annotation was used to calculate percentages of mitochondrial proteins in each set. This analysis illustrates that all crosslinked sets (being either with TwinkleFLAG or mtLucFLAG) showed approximately 70% mitochondrial annotation whereas the TwinkleFLAG versus mtLucFLAG -XL showed only 28% mitochondrial annotation. (**B**) To identify potentially interesting proteins we compared all 4 generated lists simultaneously using Venny (http://bioinfogp.cnb.csic.es/tools/venny/index.html), that generates a 4-way Venn diagram and separate lists for all intersecting and non-intersecting parts of the diagram. The region for potentially interesting proteins, being enriched with TwinkleFLAG +XL compared to respective controls is further outlined in red. The resulting list of 168, used for later comparison (see Fig. 3) is separately given alphabetically by gene name in S4 Table (first sheet: ‘Biol repeats enriched all’). S1 Table, in addition is sorted in such a way that the same 168 proteins are the first 168 proteins listed in the LFQ comparison sheet (sheet 3).

To more systematically analyse samples we next applied LTQ-FT mass spectrometry on IAP eluates, analysing the protein composition of these samples by shotgun proteomics. To optimize the procedure and establish the robustness of the crosslinking and IAP method we first measured several completely independent biological repeats over an extensive period of more than 1 year using various batches of TwinkleFLAG and mtLucFLAG cells with and without XL. Following individual sample analysis at the time of sample preparation, raw mass spectrometry data files of all samples were analysed in one batch using MaxQuant[37]. This allows for the *post-hoc* comparison of signal intensities of peptides between samples to provide a relative abundance measure for identified proteins. Based on this analysis we initially compared biological repeats by taking LFQ ratio’s for the identified proteins between the 4 conditions tested (being mtLucFLAG -XL or +XL, and TwinkleFLAG -XL or +XL), compiling lists of proteins with a least a 2-fold increase compared to its control and comparing these lists between the biological repeats. From this we extracted ‘≥2 fold increase’ lists based on the further condition that this was observed in at least 2 out of 3 samples. To finally extract meaningful protein sets we generated Venn diagrams simultaneously comparing the four generated protein lists (Fig. 2) using Venny (http://bioinfogp.cnb.csic.es/tools/venny/index.html). MaxQuant raw data output and analyses sheets can be found in S1 Table. A comparison of the enriched protein sets shows that both with Twinkle and mitochondrially targeted Luciferase, cross-linking results in a marked enrichment of mitochondrial proteins: cross-linking increased the percentage of mitochondrial proteins in both TwinkleFLAG and mtLucFLAG samples from 28 to 70% based on Gene-Ontology(GO)-SLIM annotation (Fig. 2). This analysis illustrates a considerable enrichment of mitochondrial proteins with cross-linking, suggesting the fixation of specific direct and indirect interactions with the respective baits.

**Fig 3:**
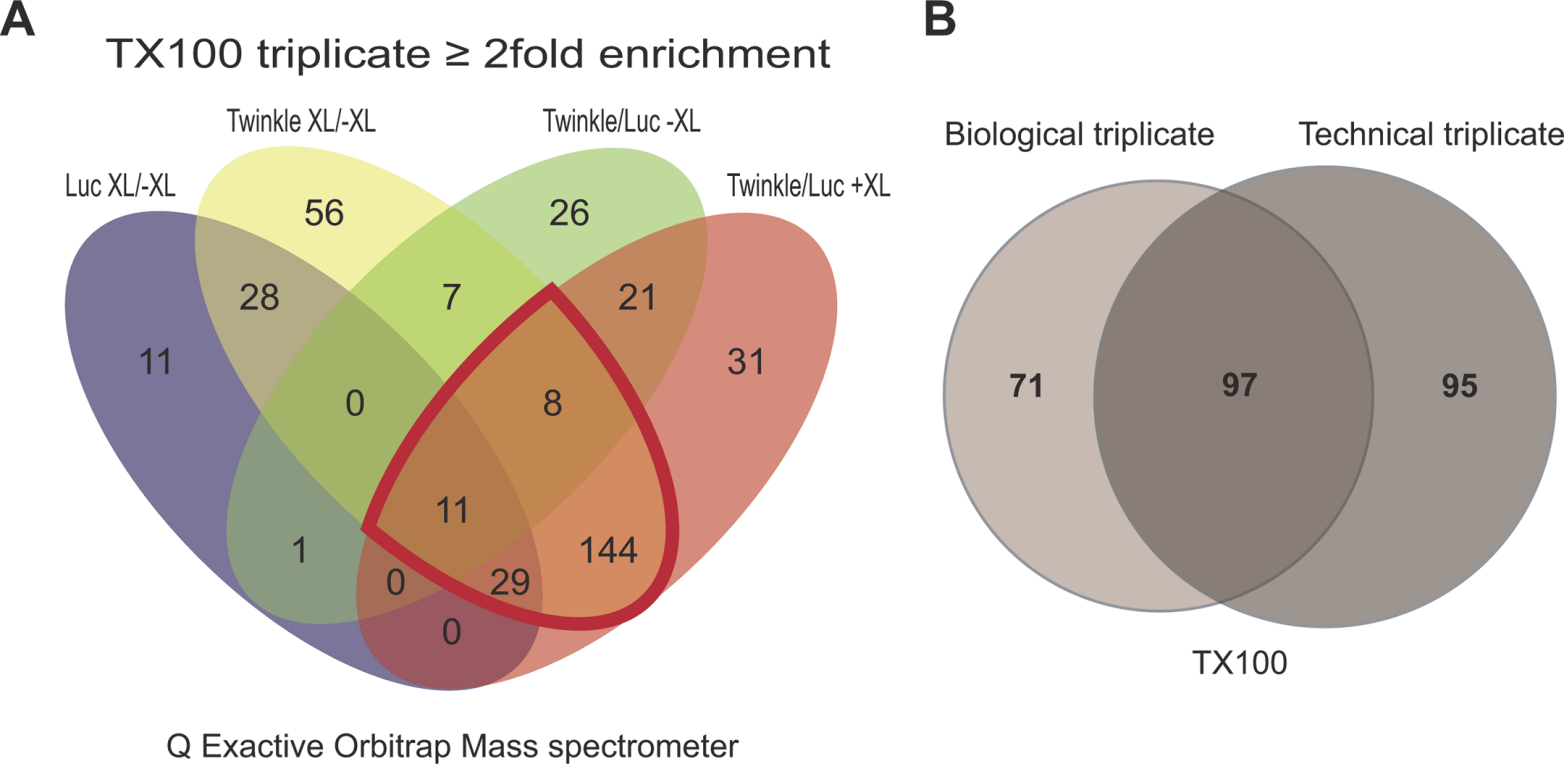
Q Exactive mass spectrometry analysis following Triton X100 based affinity purification. (**A**) Sample 2 of the 3 biological repeats (measured for Fig. 2) was measured in triplicate on a Q Exactive Orbitrap. To identify potentially interesting proteins we compared all 4 generated lists simultaneously using Venny, similar as in Fig. 2. The region for potentially interesting proteins, being enriched with TwinkleFLAG +XL compared to respective controls again is further outlined in red. The resulting list of 192, used for later comparisons (see Figs. 3B and 4) is separately given alphabetically by gene name in S4 Table (second sheet: ‘TX100 enriched all’). S2 Table, in addition is sorted in such a way that the same 192 proteins are the first proteins listed in the LFQ comparison sheet (sheet 3). (**B**) In order to compare different sets of experiments we used area-proportional Venn diagrams (BioVenn[58]). Comparing the enriched set of proteins from three biological repeats (Fig. 2) measured using an LTQ-FT mass spectrometer with series 2 of the biological repeat measurement, measured in triplicate with a Q Exactive Orbitrap mass spectrometer (see above, **A**), shows a considerable overlap between both experiments. The core set of proteins enriched in both measurements includes many established nucleoid associated proteins.

**Fig. 4:**
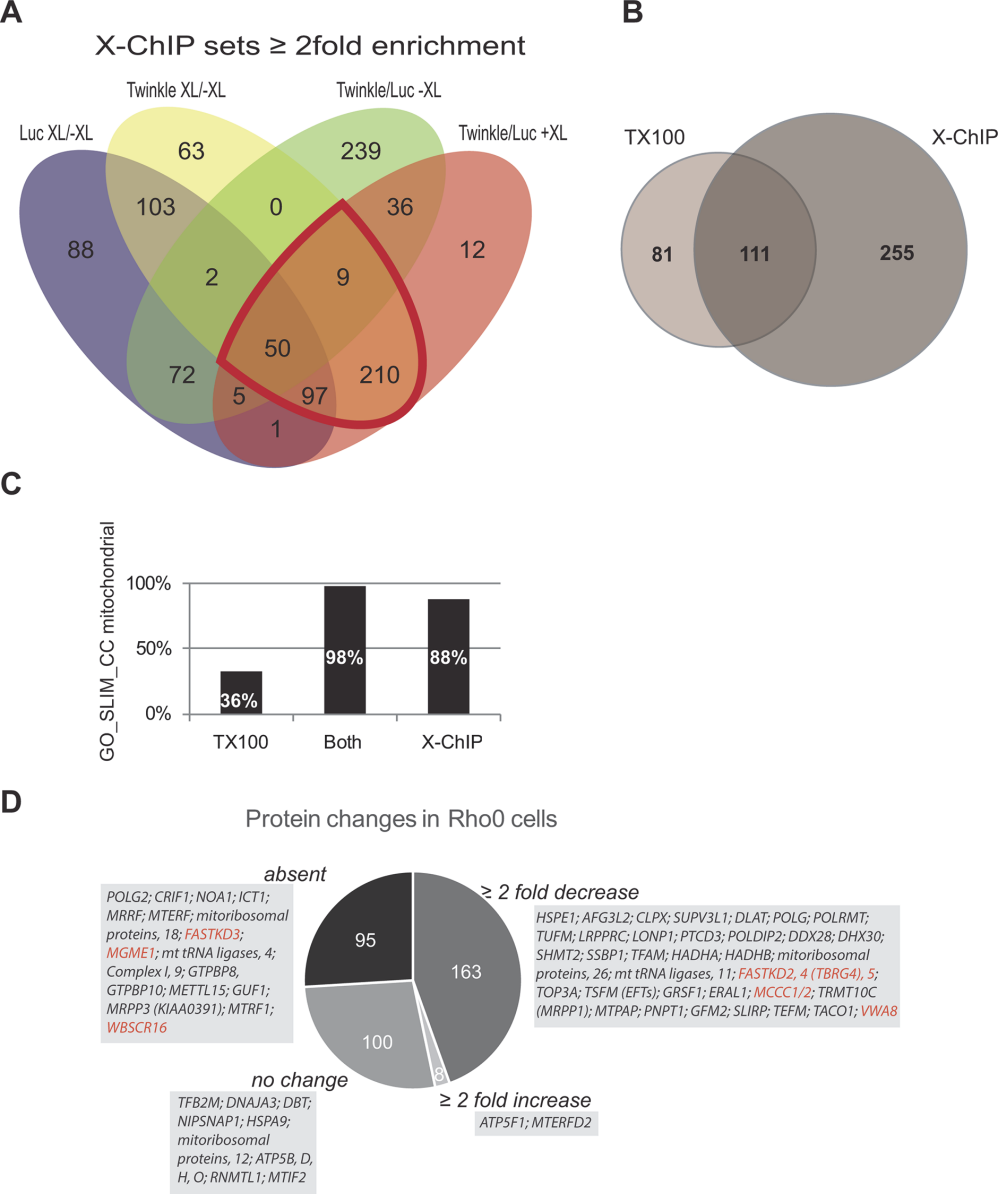
X-ChIP based affinity purification provides the most inclusive analysis of nucleoid associated proteins. (**A**) Protein complexes using FLAG-tag targeted isolation using TwinkleFLAG (Twinkle) and mtLucFLAG (Luc) cells, from cells treated either with or without FA were isolated using an X-ChIP based isolation buffer. Samples were analysed (in duplicate for TwinkleFLAG + XL, otherwise in triplicate) by shotgun mass spectrometry using a Q Exactive Orbitrap. To again identify potentially interesting proteins we compared all 4 generated lists simultaneously using Venny, similar as in Figs. 2/3. The region for potentially interesting proteins, being enriched with TwinkleFLAG +XL compared to respective controls again is further outlined in red. The resulting list of 366 proteins, used for later comparisons (see 4B/C/D) is separately given alphabetically by gene name in S4 Table (third sheet: ‘X-ChIP enriched all’). S3 Table, in addition is sorted in such a way that the same 366 proteins are the first proteins listed in the LFQ comparison sheet (sheet 3). (**B**) An area-proportional Venn diagram shows the comparison of the enriched set obtained using TX100 lysis compared to the enriched set obtained using the X-ChIP method. An analysis of the proteins identified as enriched in both sets shows that of these 111, 109 proteins (98%) have a Gene Ontology (GO)_SLIM_Cellular Compartment (CC) annotation (**C**) while the remaining 2 proteins despite the lack of such an annotation are likely also to be mitochondrial. In contrast, of the remaining 81 proteins identified as enriched exclusively with the TX100 method, only 36% is annotated as mitochondrial, while of the 255 proteins that were found specifically enriched with the X-ChIP method but absent in the TX100 dataset, 88% is annotated as mitochondrial. Again this likely is an underestimation by mis-annotation or the lack of a GO_SLIM_CC annotation. These data combined thus identify the X-ChIP method as the superior method in combination with whole cell cross-linking. (**D**) Using the X-ChIP method we now compared LFQ values of the 366 proteins obtained with regular HEK293 TwinkleFLAG cells with those obtained from HEK293 TwinkleFLAG ρ° cells. The pie-chart shown here illustrates the distribution of the 366 enriched proteins identified with the X-ChIP method in regular HEK293 TwinkleFLAG and measured in HEK293 TwinkleFLAG ρ° in the following classes: not detected (absent), 95 proteins; ≥2 fold decrease, 163 proteins; no change, 100 proteins or ≥2 fold increase, 8 proteins(see also S3 Table). Light gray boxed text shows abridged lists of proteins in each of the four categories selected from S3 Table, concentrated on proteins involved in mtDNA maintenance and gene expression and including a few other categories discussed in the text such as complex I and V, as well as a few newly identified candidate proteins. A few of the proteins that are considered novel candidate nucleoid associated proteins and that are discussed in the main text are highlighted in red. Although quite a few other proteins have not been described primarily as nucleoid associated these have been described as having a role in mitochondrial gene expression and hence have not been highlighted.

By applying a stringent comparison between experiments the most interesting lists from the perspective of identifying potential NAPs and comparing identified proteins with previously published datasets are those proteins that are enriched in TwinkleFLAG +XL compared to TwinkleFLAG -XL and mtLucFLAG XL (as marked by a red circumference in the Fig. 2 Venn diagram).

In the complete comparison of TwinkleFLAG +XL to both TwinkleFLAG -XL and mtLucFLAG +XL (168 proteins) (an annotated version is presented in S2 Table) we find a substantial number of the proteins that have been identified in various previously published datasets (see also[29]). These include core components of the mitochondrial replication and transcription machineries, such as TFAM, POLG1, mtSSB and POLRMT. Combined our analysis of several biological repeats and comparison with published datasets of NAPs (see also below), including a dataset of enriched proteins purified with the mitoribosomal associated protein ICT1 that also included many potential NAPs[29,38], shows that whole cell crosslinking in combination with IAP is a valid method to identify these proteins.

To further optimize our method to be able to more confidently identify potential novel NAPs we compared two different isolation conditions, considering that different isolation methods should yield at least a similar core set of proteins. The first is the condition used for the biological repeats above, which involves a relatively high-salt (300 mM NaCl) Triton-X100 lysis with sonication followed by IAP and washing with buffers both with high and low salt (see *Materials & Methods*). The second involves a representative protocol used for cross-linking chromatin immune precipitaton (X-ChIP) using sonication in RIPA buffer (see *Materials & Methods*) with the addition also of RNAse A, DNAse I and Benzonase since our interest is in protein analysis and not DNA analysis typical for X-ChIP. With the addition of nucleases we also hoped to more specifically identify proteins that are in close association with Twinkle and not proteins that co-purify via indirect DNA and/or RNA association (see *Discussion* & below). In addition, to give our analysis a more solid basis we measured samples as triplicate technical repeats on a Q Exactive mass spectrometer that possesses a greater sensitivity and faster MS/MS duty cycle, and again allows comparison of samples using LFQ values generated by MaxQuant.

By first measuring one of the biological repeats (sample 2) comparing TwinkleFLAG +XL, mtLucFLAG +XL versus TwinkleFLAG -XL and mtLucFLAG -XL with high-salt Triton X100 (TX100) lysis in triplicate on the Q Exactive allowed us to compare this measurement with the three biological repeats measured on an LTQ FT mass spectrometer. This showed that there is substantial overlap of enriched proteins for both sets of experiments (Fig. 3 & S3 Table) as expected. Overall, however, more proteins were identified on the Q Exactive instrument due to its greater sensitivity and faster MS/MS duty cycle. This set of measurements was now compared to a duplicate Q Exactive measurement of samples purified with the X-ChIP purification method. This comparison shows a considerable number of proteins that were identified with both methods (Fig. 4A and B), despite the presence of nucleases in the X-ChIP based purification. In fact many identified nucleoid associated proteins were detected using the X-ChIP method that were not identified using TX100 lysis. In particular a large number of mitoribosomal proteins and proteins with possible or established roles in RNA metabolism and translation, such as DDX28, TACO1, MTIF2 and MTRF1, were found. In addition, proteins that are considered nucleoid associated proteins by their demonstrated molecular function, such as POLG2 and the recently described nuclease MGME1[39–41] were specifically identified with the X-ChIP method. Possibly the X-ChIP protocol, instead of removing all proteins that are indirectly associated with TwinkleFLAG either via DNA or RNA, might result in a less tightly packed complex in turn resulting in better accessibility of the FLAG epitope for TwinkleFLAG IAP. This would explain the approximately 10-fold higher LFQ values for Twinkle with X-ChIP compared to the TX100 Q Exactive measurements, whereas mtLucFLAG LFQ values are comparable between both sets (S2 and S3 Tables). This can then be expected to result also in a much better recovery of cross-linked mitochondrial proteins in the X-ChIP experiment. Not surprisingly, 98% of all proteins enriched with both the TX100 and the X-ChiP method are mitochondrial (Fig. 4C). Moreover, 88% of proteins that are specifically enriched with the X-ChiP method had a mitochondrial annotation while in contrast, 36% of the proteins that showed specific enrichment only with the TX100 method were mitochondrial, suggesting many of these proteins are contaminants in the preparation.

We recently have shown that Twinkle helicase is firmly membrane associated and that even in the absence of mtDNA it forms discrete membrane associated foci within the mitochondrial network [17]. Based on these findings we suggested also by analogy with baker’s yeast[42] that a subset of nucleoid associated proteins might organize in a replication platform even in the absence of mtDNA. These observations could thus allow us to identify proteins associated with Twinkle in a minimal replication platform, but in addition tackle the question of indirect association via DNA/RNA binding, by purifying TwinkleFLAG following FA cross-linking using cells without mtDNA (hereafter ρ°). HEK293 FlpIn-TREx ρ° cells were established (S1 Fig.) and stable inducible TwinkleFLAG ρ° cells were subsequently generated. As ρ° cells lack mtDNA they also lack mitochondrial tRNAs as well as the two mitoribosomal RNAs and thus functional mitoribosomes cannot be assembled.

**Fig. 5:**
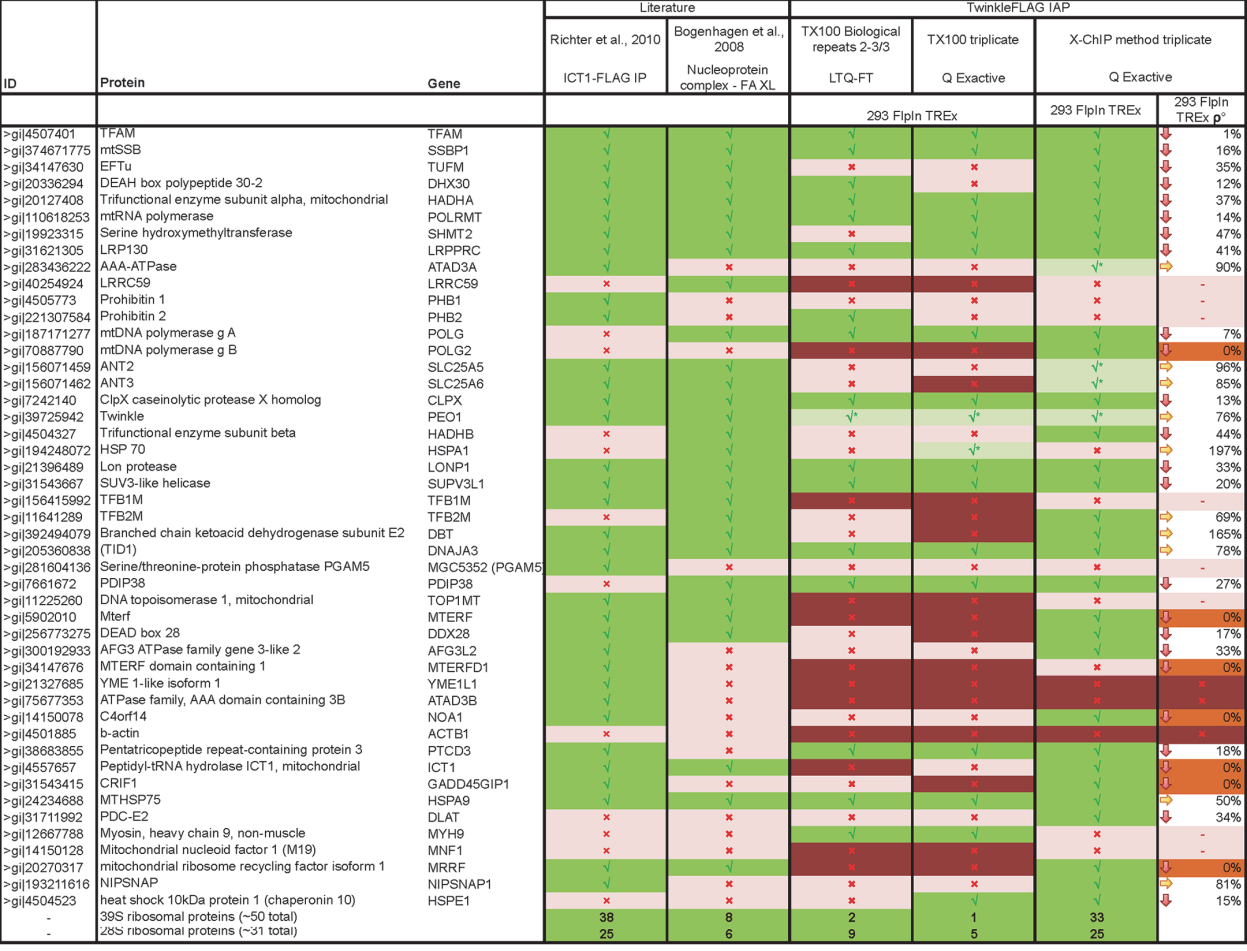
Comparing whole cell cross-linking TwinkleFLAG immune affinity purification with previous nucleoid isolations. Comparison with most commonly identified potential mtNAPs as published in[29] with their enrichment in the TwinkleFLAG +XL IAP. The data here is reduced to compare previously published mitochondrial formaldehyde cross-linking followed by nucleoid purification as performed by[22], in which for simplicity reasons both published protein list are combined to one list and the data from ICT1-FLAG IP as performed by[38]. For the full table see Hensen et al[29]. Shown are the comparison of the three biological repeats on the LTQ-FT Ultra, Q Exactive TX100 and X-ChIP method datasets. Green checkmark indicates an ≥ 2 fold increase in the TwinkleFLAG IAP compared to the mtLucFLAG IAP control with cross-linking. A light red cross indicates no difference while a dark red cross indicates undetected protein. Green checkmark indicated with an asterix represent proteins which are increased in TwinkleFLAG compared to mtLucFLAG with cross linking but not compared to non cross- linked TwinkleFLAG control (Twinkle itself is a logical representative of this class). For the ρ° samples we indicate the percentage of protein, based on LFQ ratios, co-purified in the absence of mtDNA.

Having generated lists of proteins that are enriched in TwinkleFLAG +XL compared to both TwinkleFLAG -XL and mtLucFlag +XL we now considered only those 366 proteins enriched with the X-ChIP protocol (S3 and S4 Tables) in a direct comparison of TwinkleFLAG +XL in regular HEK293 FlpIn-TREx or HEK293 FlpIn-TREx ρ° cells, each measured using the X-ChIP protocol. This revealed that 258 of 366 proteins showed a ≥2 fold decrease in ρ° cells IAP while 95 of those 258 proteins were completely absent (Fig. 4D, S3 Table). The 95 proteins that were absent in this particular ρ° TwinkleFLAG IAP included several nucleoid associated proteins on the basis of earlier demonstration of nucleoid association or a clear function in mtDNA metabolism and expression. Examples hereof include MGME1, MTERF and POLG2, while many other proteins were proteins involved in mitochondrial gene expression such as ribosomal proteins, tRNA synthetases, translation and RNA processing factors. The more inclusive list of all proteins that were reduced ≥2 fold included many additional proteins in the same categories, including for example DHX30 and DDX28, LONP1, GRSF1, POLRMT and so on. The ρ° TwinkleFLAG IAP results point to proteins that co-purify with TwinkleFLAG in regular HEK293 FlpIn-TREx cells by means of association with DNA/RNA or possibly other higher order structures that are modified or absent in ρ° cells. A comparison of commonly identified proteins associated with purified cross-linked nucleoids and mitochondrial ribosomes[29], proteins purified using our two isolation methods and cell lines either with or without DNA is given in Fig. 5. This figure again illustrates not only that with the X-ChIP protocol we identify the majority of previously identified proteins but also how these proteins change in ρ° cells.

As pointed out above, those proteins that are not changed more than 2-fold or that are increased might also be of interest. Similar protein levels might indicate nucleoid associated proteins that directly interact with TwinkleFLAG or with a membrane platform and/or a minimal nucleoid that is still present in ρ° cells and that Twinkle is part of[17]. These proteins (see Fig. 4D, S3 Table) include for example ATAD3, MTERFD2 and ATP synthase subunits (see *Discussion*).

## Discussion

In this paper we demonstrate the feasibility of using whole cell formaldehyde cross-linking in combination with immuno-affinity purification and tandem mass spectrometric analysis in the identification of a mitochondrial protein complex, in this case the nucleoid mtDNA-protein complex. We show that this method identifies many of the same proteins as previously published mitochondrial FA cross-linking experiments combined with several subsequent more laborious purification steps. The method in addition identified several novel proteins that should be considered prime candidate nucleoid associated proteins. By the application of label free quantification we could analyse the effects of isolation buffers and the effect of isolating Twinkle in the absence of mtDNA and consequently all mitochondrially encoded RNAs. The latter experiment was very revealing in that it identified many proteins that were considerably reduced or absent in TwinkleFLAG IAP from ρ° cells pointing to their association with the nucleoid in mtDNA containing cells on the basis of DNA/RNA association and notwithstanding the possibility that a number of these proteins might also be less stable in the absence of mtDNA/RNA. Many of these proteins should thus be considered as nucleoid associated. In addition it pointed to at least some proteins that are found in close vicinity or directly interact with Twinkle and could form part of a minimal membrane associated platform. Combined our results position the mitochondrial gene expression machinery including proteins involved in RNA processing and translation in close vicinity to nucleoids while at the same time providing an important resource for mtDNA maintenance and gene expression machinery protein discovery.

### Whole cell cross-linking combined with IAP can be applied to mitochondrial protein complexes

Because of its small size, formaldehyde is able to enter cells rapidly and efficiently, cross-link proteins and freeze even transient interactions[43]. In the case of mitochondrial protein cross-linking this has the advantage that mitochondrial proteins can be cross-linked to other proteins and nucleic acids with very little disturbance of the native environment. An additional advantage of formaldehyde is that the cross-link is reversible. Potential formaldehyde-induced protein modifications were not observed by us following heat-induced reversal of the cross-link, allowing for efficient mass spectrometry based analysis of protein samples. Formaldehyde cross-linking is not considered to be specific in literature, which might result in many false positives. By applying stringent analysis criteria, the use of various controls and a combination of cross-linking and IAP we show here that we nevertheless most consistently identified mitochondrial proteins that are furthermore considerably enriched when we compare cross-linked with non cross-linked samples. This was especially the case using the X-ChIP protocol. The use of a FLAG epitope tag poses another potential problem[43] as the FLAG tag contains several lysines that are substrates for FA cross-linking, but we have shown here, both by Western blot analysis and by LFQ-based quantitative mass spectrometry, that in our hands the combination of a short formaldehyde exposure in whole cell crosslinking did not result in dramatic adverse effects on the efficiency of FLAG IAP. This is very important as it shows that we can directly compare -XL with +XL conditions. Likewise comparison of LFQ values for TwinkleFLAG between regular HEK293 FlpIn-TREx or HEK293 FlpIn-TREx ρ° show only a 24% lower level in the ρ° cell IAP showing the validity of the comparison of LFQ values of co-precipitated proteins. The analysis presented here thus shows that our approach can have a much wider application in the analysis of mitochondrial protein complexes.

### Can we define a consensus list of nucleoid associated proteins based on formaldehyde cross-linking?

Formaldehyde can cross-link proteins to nucleic-acid but more efficiently cross-links proteins to proteins. Combine this with a high mitochondrial protein density and the tremendous sensitivity and speed of modern mass spectrometers, which is also illustrated here by the considerable increase of identified proteins by the use of a Q Exactive Orbitrap compared to a LTQ-FT mass spectrometer, and the answer to the above question clearly is no. What we do show here however, similar to what was recently discussed [29], is that formaldehyde cross-linking in combination with an appropriate isolation method yields an inclusive list of proteins, proteins that in addition might be found in close vicinity to the nucleoid in what could be considered a mitochondrial nucleoid ‘compartment’. This compartment, similar to earlier suggestions[22,24,26,44], would contain not only mtDNA and associated factors but also the many proteins involved in mitochondrial RNA metabolism and translation. In fact a large fraction of proteins we have identified as ‘nucleoid’-enriched fall in this last category, as also found by He et al[24]. This nevertheless does not discredit our method to identify potential nucleoid associated proteins if the translation and RNA processing machinery is nucleoid associated, as recent papers indeed have suggested[24,26]. However, as we have shown here, it is important to apply a systematic analysis, optimizing the condition of isolation, using various controls such as a tagged and mitochondrially targeted Luciferase, measuring both biological and technical repeats and applying stringent selection criteria in a comparative proteomics approach. This approach has for example shown that a number of proteins can be categorized as consistently enriched (Fig. 5), including many proteins that based on other research has pinpointed them as nucleoid-associated. Our approach has also shown that by a comparison of lysis conditions and sample handling (TX100 or X-ChIP), the X-ChIP method was the most sensitive and inclusive. Despite the fact that many more proteins were ‘nucleoid’ enriched compared to the TX100 method, the X-ChIP method showed enrichment of the highest percentage of mitochondrial proteins suggesting the method nevertheless is considerably more stringent than the TX100 method. Nucleoid associated proteins that only were identified using the X-ChIP method include, MGME1, DDX28, MTERF and MTERF2, Topoisomerase 3α, POLG2, TFB2M as well as 50 mitoribosomal proteins and a considerable number of other proteins of mitochondrial gene expression. To immediately assign novel candidates that are likely core nucleoid proteins with a function in mtDNA metabolism is difficult on the basis of our results, but based on the fact that many mtDNA maintenance proteins are among the proteins identified suggests that various candidates with no current assigned role in mtDNA metabolism are present among the remaining proteins. Other isolation methods that more directly probe the interaction of proteins with mtDNA could in the future more specifically identify those proteins. If we examine the data from a more holistic point of view, we can expect that a number of proteins with an as yet unassigned role in mitochondrial gene expression, including RNA metabolism and translation, are amongst the enriched proteins. The analysis of proteins that are ≥2 fold reduced or completely absent in cross-linked TwinkleFLAG IAP from ρ° cells further identifies some of these candidates. These include 4 FAST kinase domain-containing proteins (S3 Table) that were recently also identified in a published RNA-binding proteome[45]. A recent analysis of 107 proteins with a possible function in mitochondrial RNA processing also identified FASTKD4 as being involved in mRNA stability[46]. Of the 107 proteins analyzed in this paper 47 are identified in our set of 366 proteins enriched in TwinkleFLAG cross-linked samples, while 34 of these 47 proteins are ≥ 2-fold reduced in TwinkleFLAG IAP from ρ° cells. Our data provide a valuable additional resource for identification of further mitochondrial RNA metabolism proteins. One possible example is methylcrotonoyl-CoA carboxylase, an enzyme involved in leucine breakdown and to our knowledge not previously identified as nucleoid associated. Interestingly, a second enzyme in the leucine breakdown pathway, enoyl-coenzyme A (CoA) hydratase with AUUU RNA binding activity (AUH), was recently shown to reside in the mitochondrial inner-membrane and matrix and possess a function in mitochondrial protein synthesis[47] and according Wolf and Mootha also has an RNA processing phenotype[46]. AUH in our dataset was specifically enriched in TwinkleFLAG IAPs but was equally enriched without or with cross-linking. Furthermore, it was not substantially decreased in TwinkleFLAG IAP from ρ° compared to IAP from mtDNA-containing TwinkleFLAG expressing cells, suggesting this protein might be one of several proteins that more specifically interacts directly with Twinkle or is part of a Twinkle-containing membrane platform. Apart from proteins with known functions that might have adopted additional functions, such as AUH, our dataset also contains several proteins of unknown function that might be worth investigating including von Willebrand factor A domain-containing protein 8 (VWA8) and Williams-Beuren syndrome chromosomal region 16 protein (WBSCR16), both of which have a very high mitochondrial localization prediction. WBSCR16 was, similar to the FASTKD proteins, also identified in recently published RNA binding proteomes as were many other known mitochondrial RNA binding proteins [45,48].

Few proteins have been shown to have a role in nucleoid membrane attachment. We showed recently that Twinkle organises replicating nucleoids to the inner mitochondrial membrane compartment and that Twinkle remains associated to the membrane in discrete foci in ρ° cells[17]. In other work Prohibitin (PHB) and ATAD3 have been isolated with nucleoids and been postulated to have an architectural role in nucleoids[21,24,49]. He and co-workers showed ATAD3 and PHB to co-sediment and co-purify with nucleoids and the mitochondrial translation machinery, postulating that ATAD3 links mitochondrial ribosomes to nucleoids and that both Prohibitin and ATAD3 link nucleoids to the inner mitochondrial membrane. This was recently further corroborated using complexome profiling, showing that a substantial number of proteins of the small ribosome subunit, ATAD3A and PHB1/2 co-migrate in Blue-native gels[34]. Although PHB1 and 2 did not pass our selection criteria, because they were also identified in TwinkleFLAG IPs without XL and were not sufficiently enriched in TwinkleFLAG +XL compared to mtLucFLAG +XL, their levels remained equal in TwinkleFLAG +XL IAP in ρ° cells compared to mtDNA containing cells. ATAD3 also just failed to pass our selection criteria as it showed a <2 fold (1,93) increase comparing TwinkleFLAG +XL and TwinkleFLAG without XL. These results thus maintain the notion that these proteins could be part of a membrane anchor for a minimal mtDNA replication platform that includes Twinkle. Interestingly the X-ChIP method also identifies a number of ATP synthase subunits being enriched in TwinkleFLAG IAP while remaining constant or increasing in TwinkleFLAG IAP from ρ° cells, in contrast to subunits of for example Complex I that were mostly ≥ 2-fold reduced or absent. A recent RNAi screen for proteins with a possible role in nucleoid organization and mtDNA maintenance in *Drosophila* identified most of the nuclear ATP synthase subunits[50]. Given the involvement of ATP synthase in mitochondrial membrane organization[51–53], the combined results suggest that ATP synthase could also be involved in the membrane organization of Twinkle containing complexes.

Previous nucleoid research has clearly pointed towards nucleoids being complex dynamic structures that have more functions than only being replication machineries. Bogenhagen *et al*[22] discusses the nucleoid structure to be layered and He et al[24] points towards an intimate relationship between nucleoids and the protein synthesis machinery, as also previously suggested by Iborra on the basis of fluorescent microscopy analysis[44]. In addition, in a recent paper Bogenhagen et al present evidence that initial RNA processing and ribosome assembly takes place in the close vicinity of nucleoids[26], whereas others have suggested that the entire small subunit of the mitochondrial ribosome is assembled at the nucleoid (see[25] and above[34]). This was further substantiated by a recent study that showed that failure to form the monosome prolongs the association of the 28S subunit with the nucleoid leading also to mtDNA aggregation[54]. Our comparison of proteins purified with TwinkleFLAG in HEK cells and their ρ° counterparts indicates that some of the proteins suggested by Bogenhagen (in particular of the small ribosomal subunit; S7, S9 and S15) to associate with nucleoids to facilitate the early steps in ribosome biogenesis, to be less than 2-fold decreased suggesting these proteins might maintain a stable association with a minimal nucleoid structure also in the absence of mtDNA and RNA. A less than 2-fold decrease was also observed for some proteins that might facilitate ribosome biogenesis such as RNMTL1[55,56] and early steps in translation such as MTIF2.

To summarize, we here show that whole cell cross-linking in combination with IAP and appropriate lysis conditions enriches for mitochondrial nucleoids and associated proteins. This method is much less elaborate and complicated compared to previously published isolation protocols that include a formaldehyde cross-linking step. Whole cell cross-linking followed by IAP results in an inclusive list of enriched proteins that we show by the use of appropriate controls and cells lacking mtDNA to contain known and candidate mtDNA maintenance proteins and factors that are involved in mitochondrial gene expression. Our method and data therefore provide a valuable tool and resource for mitochondrial researchers. Our results add further weight to the idea that mtDNA nucleoids are an important organizing centre for mitochondrial biogenesis that might even include a local and specialized membrane organization in a ‘microcompartment’, as recently suggested[57].

## Supporting Information

S1 Fig Total depletion of mtDNA in ρ° HEK293 Flp-In T-Rex cells. DNA was extracted from cells, digested with *Pvu*II and imaged by exposure to Ultra-Violet (UV) light, or blotted and probed for mtDNA and exposed to a phosphor screen for two hours (2 hour) or 16 hours (16 hour) respectively. (A) 1 kb DNA Ladder. (B) Un-treated HEK293 Flp-In T-Rex cells total DNA. (C) Ethidium bromide-treated HEK293 Flp-In T-Rex cells total DNA at 95days. (D) Ethidium bromide treated HEK293 Flp-In T-Rex total DNA at 116 days.
(EPS)

S1 Table TX100 Biological repeats. Data file of the comparison of the three biological repeats measured on the LTQ-FT generated by MaxQuant. In sheet 1 (“RAW”) the raw data MaxQuant analysis output is shown with two separate sheets showing the corresponding peptide count per protein (sheet 2; “Peptides”) and the LFQ values with their calculated ratios across samples (sheet 3; “LFQ ratio”). Per experiment the ratios were calculated and shown with arrows if there was a change observed (green arrow up, ≥ 2 fold increase; yellow arrow horizontal, no change; red arrow down, ≥ 2 fold decrease). Whenever an increased was observed in at least two out of the three experiments, this was indicated with a green checkmark (instead of a red cross when this was not observed. Please note that all LFQ values of 0 have been replaced by 1E-12 to avoid division by 0.
(XLSX)

S2 Table TX100 Q Exactive triplicate. Data file of the comparison of the three technical repeats of the samples prepared with the TX100 method measured on the Q Exactive generated by MaxQuant. In sheet 1 (“RAW”) the raw data MaxQuant analysis output is shown with two separate sheets showing the corresponding peptide count per protein (sheet 2; “Peptides”) and the LFQ values with their calculated ratios across samples (sheet 3; “LFQ ratio”). To calculate the LFQ ratio, first the average is calculated from the three technical repeats. Whenever a protein was not identified in every single repeat, it was not considered (shown separately sorted on Twinkle-FLAG occurrence). Next to the ratio it is indicated if a change was observed (green arrow up, ≥ 2 fold increase; yellow arrow horizontal, no change; red arrow down, ≥ 2 fold decrease). Please note that all LFQ values of 0 have been replaced by 1E-12 to avoid division by 0.
(XLSX)

S3 Table X-ChIP method Q Exactive triplicate. Data file of the comparison of the three technical repeats of the samples prepared with the X-ChIP method measured on the Q Exactive generated by MaxQuant. In sheet 1 (“RAW”) the raw data MaxQuant analysis output is shown with two separate sheets showing the corresponding peptide count per protein (sheet 2; “Peptides”) and the LFQ values with their calculated ratios across samples (sheet 3; “LFQ ratio”). To calculate the LFQ ratio, first the average is calculated from the three technical repeats (for technical reasons TwinkleFLAG + XL is only represented by 2 repeated measurements). Whenever a protein was not identified in every single repeat, it was not considered (shown separately sorted on Twinkle-FLAG occurrence). Next to the ratio it is indicated if a change was observed (green arrow up, ≥ 2 fold increase; yellow arrow horizontal, no change; red arrow down, ≥ 2 fold decrease). The table is further sorted so that the enriched TwinkleFLAG + XL set of 366 proteins as indicated in Fig. 4 are listed first, further sorted by their level detected in TwinkleFLAG + XL IAP from ρ° cells, as follows from top to bottom: 95 proteins not detected in ρ° cells, 163 proteins with a ≥ 2-fold decrease, 8 proteins with a ≥ 2-fold increase, and 100 proteins with no change (< than 2 fold change). Please note that for calculation purposes all LFQ values of 0 have been replaced by 1E-12 to avoid division by 0.
(XLSX)

S4 Table Datasets of enriched proteins including annotations. Enriched proteins from 4-sample Venn diagrams depicted in Figs. 2–4 and demarked by a red circumference are listed here in alphabetical order by Gene Symbol (first 3 sheets). These datasets were used to generate the area-proportional Venn diagrams in Figs. 3B and 4B and associated protein lists, comparing i) the 3 biological (Biol) repeats measured on and LTQ-FT (FT) with a triplicate measurement of sample 2 (Biol 2) of the three biological repeats measured on a Q Exactive (QE) mass spectrometer (sheets: Biol FT & Biol 2 (TX100) QE, 97 proteins; Biol FT NOT QE, 71 proteins; Biol 2 FT NOT QE, 95 proteins) and ii) the Biological repeat 2 Triton X100 based method with the X-ChIP based purification method both measured on a Q Exactive instrument (sheets: TX100 & X-ChIP, 111 proteins; TX100 NOT X-ChIP, 81 proteins; X-ChIP NOT TX100, 255 proteins).
(XLSX)
